# Effects of *Bacillus subtilis* iturin A on HepG2 cells in vitro and vivo

**DOI:** 10.1186/s13568-021-01226-4

**Published:** 2021-05-10

**Authors:** Haobin Zhao, Lu Yan, Ling Guo, Hui Sun, Qingsheng Huang, Dongyan Shao, Chunmei Jiang, Junling Shi

**Affiliations:** 1 Key Laboratory for Space Bioscience and Biotechnology, School of Life Sciences, Northwestern Polytechnical University, 127 Youyi West Road, Xi’an, 710072 Shaanxi China; 2 School of Hospitality Management, Yanshan District, Guilin Tourism University, 26 Liangfeng Road, Guilin, 541006 Guangxi China

**Keywords:** *Bacillus subtilis*, Iturin A, HepG2, Proliferation, Immune microenvironment, Physiological toxicity

## Abstract

Iturin A with cyclic peptide and fatty acid chain isolated from *Bacillus subtilis* fermentation shows a variety of biological activities. Among them, the anticancer activity attracted much attention. However, the molecular mechanism of its inhibitory effect on hepatocellular carcinoma was still unclear. Thus its effect on hepatocellular carcinoma was tested in this research. It was found that iturin A could enter HepG2 cells immediately and cause reactive oxygen species burst, disrupt cell cycle and induce apoptosis, paraptosis and autophagy in vitro. The iturin A without fatty acid chain showed no antitumor activity. Amphiphilic is critical to the activity of iturin A. The anticancer activity of iturin A to hepatocellular carcinoma was also verified in mice models carrying xenograft tumors constructed by HepG2 cells. At a dosage of 3 mg/kg/day, iturin A significantly inhibited the further increase of the tumor weight by 58.55%, and reduced the expression of Ki67 in tumor. In the tumor treated with iturin A, lymphocyte infiltration was found, and the expressions of TGF-β1and PD-L1 were decreased, which indicated that the tumor immune microenvironment was improved. Besides, iturin A showed no significant harm on the health of mice except slight disturbance of liver function. These results suggested that iturin A had significant antitumor effect in vitro and vivo, and provide a basis for the application of iturin A as anticancer agent.

**Supplementary Information:**

The online version contains supplementary material available at 10.1186/s13568-021-01226-4.

## Key points

- Depending on its amphiphilic, *Bacillus subtilis* iturin A could enter HepG2 cells rapidly, and induce apoptosis, autophagy and paraptosis in vitro*.*
- Iturin A could significantly inhibit the growth of xenograft tumor in mice with the tumor growth inhibition rate nearly 58.55%. And iturin A showed no significant harm on the health of mice except slight effect on liver function.
- Iturin A decreased the expressions of TGF-β1 and improved the tumor immune microenvironment.

## Introduction

Hepatocellular carcinoma (HCC) is one of the major malignancies worldwide and is the second-leading cause of cancer-related death globally (Chan and Ng 2020; Zucman-Rossi et al. 2015). Many materials have been used to treat HCC, such as doxorubicin, cisplatin, 5-fluorouracil, doxorubicin, interferon, sorafenib and a number of other agents (Bruix et al. 2019). However, because of low drug response rates, the clinical use is limited (Kudo et al. 2018). Thus, it is critically needed to explore safe and efficient drugs for the therapy of HCC.

The diversity of bioactive molecules in microbial metabolites provides a rich source of natural products for the exploration of new drugs. *Bacillus subtilis* is a nonpathogenic bacterium widely distributed in environment. Due to its abundant bioactive metabolites, *B. subtilis* is widely used in food preservation, agriculture, animal husbandry, medicine and so on(Guo et al. 2017; Jiang et al. 2014; Lee et al. 2019; Zohora et al. 2016). Lipopeptides from *B. subtilis* have been proved to have antifungal, antiviral, antitumor and other biological activities. Due to the composition of hydrophobic fatty acids and hydrophilic peptides, lipopeptides have the ability to decrease surface and interfacial tension of biofilms. This may be the source of lipopeptide bioactivity (Zhao et al. 2017). Iturin A is one of the lipopeptides produced by *B. subtilis*. It has been previously found to have significant antifungal activities and was proved to inhibit fungal contamination in fruits and during wine processing(Jiang et al. 2014 , 2017), thus showing potential in preventing fungal contamination in food protection and food processing (Meena and Kanwar 2015). Besides, animal experiments in mice showed that oral administration of iturin was safe and beneficial to the health of mice. The results showed that the content of cholesterol in blood was decreased and the abundance of probiotics in intestinal flora was increased (Zhao et al. 2018a). Among the multiple bio-activities of iturin A, anticancer capability is also very attractive, which has been proved in cancer cell lines, including MDA-MB-231, MCF-7 and Caco2. With regard to the anticancer mechanism, iturin A can induce apoptosis in cancer cells by triggering both reactive oxygen species (ROS) burst and DNA damage. It can also induce autophagy and paraptosis, resulting in metabolic disorder and programmed cell death (Dey et al. 2015b; Zhao et al. 2019a). Although there have been several researches on anticancer activity of iturin A, the effect of iturin A on HCC hasn’t been proved in vivo.

In this research, the potential of iturin A to inhibit the HCC cell line HepG2 was tested both in vitro and in vivo. The underlying mechanisms and toxicity were also analyzed.

## Materials and methods

### Preparation of iturin A from Bacillus subtilis culture

The iturin A was prepared from a culture of *Bacillus subtilis* CCTCCM207209(Accession number of 16S rRNA Gene sequence in GeneBank: MT373810) (Yan et al. 2020), a strain previously isolated from soil and stored at the China Center for Type Culture Collection (Wuhan, China) according to a previously published method(Zhao et al. 2018b). The lipopeptides were isolated from the supernatant of the culture broth with *B. subtilis* filtered out. Purification of iturin A-like lipopeptides was conducted using methanol extraction and HPLC isolation as well as purification in a Shimadzu LC-20A high-performance liquid chromatography (HPLC) (Shimadzu, Japan) system quipped with a C18 column and detection was made by determining absorbance at 280 nm and 215 nm. Results of electrospray ionization mass spectrometry (ESI–MS) and nuclear magnetic resonance (NMR) indicated that the purified fraction was iturin A with m/z = 1043.5 Da and composed of seven amino acids and 3-amino-tetradecanoic acid. It is a ring structure composed of amide bonds (Asn → D-Tyr → D-Asn → Gln → Pro → D-Asn → Ser → β-amino fatty acid with a-(CH_2_)_10_CH_3_ group as a side chain) (Jiang et al. 2020).

### *Anticancer potential of iturin A against HepG2 *in vitro

#### Cell culture and cell proliferation assay

The HCC cell line HepG2 were obtained from the cell bank of the Typical Culture Preservation Committee of the Chinese Academy of Sciences (Shanghai, China). Cells were cultured in DMEM (Hyclone, UT, USA), supplemented with 10% fetal bovine serum (FBS). Cells were maintained in humidified incubator at 37 °C and 5% CO_2_.

Cells were seeded in 96 wells plate at a density of 1 × 10^4^ cells per well. After 4 h, iturin A was added to the cell culture at different working concentrations of 5 µM, 10 µM, 25 µM, 50 µM, 75 µM, and 100 µM, respectively. After 48 h, MTT solution was added to the 96-well plate at 10 µL per well. After 4 h of incubation at 37 °C, the supernatant was removed and dimethyl sulphoxide (DMSO) was added. The cell viability was determined at 490 nm with a microplate reader (BioTek, VT, USA). Cell viability was calculated using Formula (1). The control group was considered as 100% (Almutary and Sanderson 2016).

1 $$Cell\,viability = \frac{{\left( {A_{sample} - A_{blank} } \right)}}{{\left( {A_{control} - A_{blank} } \right)}} \times 100\%$$

### Visualization of cell morphology and ultrastructure

Cells were seeded in 6-well plates at a density of 1 × 105 cells per well. Iturin A was added into the medium to treat HepG2 cells for 48 h. In control, same volume of solvent (DMEM) was added to the medium. After incubation, the cells were observed via optical microscopy (Nikon 80i, Japan).

Transmission electron microscopy (TEM) was used to watch the ultrastructure of cells. After being washed with PBS, cells were fixed in 2.5% glutaraldehyde for 2 h at 4 °C. Then the cells were subjected to gradient dehydration with ethanol, replacement of ethanol with tertiary butanol, embedded with resin, polymerization, and section, and stained with uranyl and lead acetates. Samples was watched and captured with transmission electron microscope Hitachi H-7650.

### Apoptosis detection by flow cytometry

The percentage of apoptotic cells was quantified with the Annexin V-FITC Apoptosis Detection Kit (Beyotime, Nanjing, China) by flow cytometry. Cells from either control group or treated group were harvested and washed with PBS. According to the manufacturer’s instructions, cells were resuspended in 195 µL of binding buffer, 5 µL annexin V-FITC and 10 µL PI were added, and the mixture was incubated for 15 min in the dark at room temperature. Analysis was performed on a flow cytometer (BD, NY, USA) (Xu et al. 2017).

### Cell cycle analysis using flow cytometry

The distributions of cells in the different phases of the cell cycle were also measured by flow cytometry. According to the DNA content at different periods, the cell proportion at different periods can be distinguished. Firstly, HepG2 cells were treated with iturin A for 48 h. After this treatment, the cells were washed with ice-cold PBS and fixed in cold 70% ethanol overnight at 4 °C. Then, cells were washed with PBS and resuspended in PBS containing PI (10 µg/mL) and RNase A (50 µg/mL). After incubation for 30 min (at 37 °C) in the dark, analysis was performed via flow cytometry (BD, NY, USA).

### Measurement of reactive oxygen species

To monitor the reactive oxygen species (ROS) levels, cells were treated with iturin A for 48 h, washed with PBS, harvested, loaded with 10 µM DCFH-DA (Beyotime, Nanjing, China) for 20 min at 37 °C, and then washed with DMEM. The levels of intracellular ROS were represented by the fluorescence intensity of 2,7-dichlorofluorescein (DCF). At last, the cells were checked and captured under a fluorescence microscope (Nikon 80i, Japan), and fluorescence intensity was detected (Zhao et al. 2019b).

### Analysis on the capability of iturin A to enter cells

To track whether iturin A can enter cells, the fluorescent agent 5(6)-Carboxy fluorescein N-succinimidyl ester (CFNSE) was used to label iturin A. The amide bond of CFNSE can be broken by organic solvents, and then react with the free amino group in the polypeptide to form amide bond again. In this way, CFNSE can be combined with iturin A. In operation, 5 mg of CFNSE was mixed with 10 mg purified iturin A in a 15 mL centrifuge tube containing 10 mL chloroform. After incubation at 20 °C overnight, the mixture was transferred to a 50 mL centrifuge tube with 10 mL ultrapure water. After shaking and mixing, the aqueous phase was detected and purified using a silica gel plate with the mobile phase of ethyl acetate after a 5 min of centrifugation at 10,000 rpm. The fluorescein-labeled iturin A was separated and purified by thin-layer chromatography (TLC). The purified CFNSE-iturin A was dissolved in cell culture medium and incubated with HepG2 cells for 10 min, and then observed under a fluorescence microscope and photographed. The control group was cells incubated with CFNSE for the same time.

### Effect of peptide in iturin A to HepG2 cells

To verify the effect of peptide on the activity to HepG2 cells, the peptide (Asn → Tyr → Asn → Gln → Pro → Asn → Ser) in iturin A was synthesized (ChinaPeptides Co. Shanghai, China). Analysis activity of peptide to HepG2 cells was done as method previous (Zhao et al. 2018b). 4 h after HepG2 cells were seeded, the peptide was added to the cell culture at different working concentrations of 5 µM, 10 µM, 25 µM, 50 µM, 75 µM, and 100 µM, respectively. After 48 h, the cell viability was detected by MTT assay and calculated using Formula (1). The control group was considered as 100%.

### Anticancer potential of iturin A against HepG2 in vivo

#### HCC xenograft model in mice

Female, 4-week-old BALB/c nude mice were purchased from Shanghai SLAC Laboratory Animal Company, Ltd. (Shanghai, China). The mice were reared in the SPF animal house. Human hepatocellular carcinoma HepG2 cells used for implantation were harvested when they were vigorous, and were resuspended in DMEM medium at a concentration of 1 × 10^7^ cells/mL. 100 µL of the prepared cell suspension was subcutaneously inoculated into the right axilla of nude mice.

When the tumor volume reached 100 mm^3^, the mice were randomly divided into two groups, and iturin A treatment was administered. The iturin A treatment group received daily iturin A treatment at a dosage of 3 mg/kg (body weight) per day. After 15 days, mice were sacrificed, and the serum, liver, kidney, and spleen were collected. Xenograft tumors were collected and their diameters were measured with Vernier calipers. Tumor volume was calculated using Formula (2):

2 $$Tumor\,volume\, = \,\frac{1}{2} \times 1 \times w^{2}$$

where *w* represents the width and l represents the length (mm) of the tumor tissue. The tumor volume was measured with calipers. The tumor relative proliferation rate (RPR) was calculated using Formula (3):

3 $$RPR\left( \% \right)\, = \,\frac{{T_{RTV} }}{{C_{RTV} }}\, \times \,100\%$$

where *T*_*RTV*_ and *C*_*RTV*_ represent the relative tumor volumes in the iturin A treatment group and the control group, respectively.

The inhibition percentage of tumor growth was calculated using Formula (4):

4 $$TGI\left( \% \right)\, = \,1 - \frac{{m_{t} }}{{m_{c} }}\, \times \,100\%$$

where *m*_*t*_ and *m*_*c*_ represent the average tumor weight of the iturin A treatment group and the control group, respectively.

### Detection of intracellular ROS levels in tumor tissue

Tumor tissue was fixed for more than 24 h in fixation fluid and then dehydrated in sucrose solution. After embedding and slicing, the tissue was dyed with ROS dying solution, detected with fluorescence microscopy, and photographed.

### Caspase-9 and caspase-3 activity assays of tumor tissue

Caspase-3 and caspase-9 activities were detected by using the Caspase-3/Caspase-9 Activity Assay Kit (Beyotime, Nanjing, China). The activity was measured as fluorescence generated by the generation of yellow p-nitroanilide (pNA), which was generated by the addition of an appropriate peptide substrate. Firstly, the tumor tissue was lysed on ice. The supernatant of the cell lysate was collected, and mixed with buffer containing acetyl-Asp-Glu-Val-Asp p-nitroanilide (Ac-DEVD-pNA) as a substrate peptide, which was followed by incubation at 37 °C for 3 h. The pNA release was quantified by measuring the absorbance at 405 nm with a microplate reader (Bio-Tek, VT, USA). The concentration of pNA was calculated form the pNA standard curve. One unit is the amount of enzyme that will cleave 1.0 nmol of the colorimetric substrate Ac-DEVD-pNA per hour at 37 °C under saturated substrate concentrations. The total protein concentration was measured by the Bradford method. The specific Caspase-9/3 activity was normalized to the total protein and then expressed as a fold increase or decrease of the Caspase-9/3 activity compared with the control group (Zhao et al. 2015).

### Immunohistochemistry analysis of tumor tissue

Fresh tumor tissues were immediately fixed in 4% paraformaldehyde solution, embedded in paraffin, and stained with hematoxylin and eosin (H&E) for morphological examinations. Serial sections were cut at 4 µm thickness, heated in an oven at 60 °C for 2 h, and subjected to immunohistochemical staining using monoclonal antibodies of Ki67, PDL-1 and TGF-β1. In operation, the tumor tissue sections were deparaffinized and rehydrated in xylene and graded alcohol. The sections were then incubated with 0.3% H_2_O_2_ in methanol for 20 min to block endogenous peroxidase activity. After blocking non-specific proteins with a normal goat serum blocking solution, the sections were incubated with antibody for 24 h at 4 °C. The sections were then washed and incubated with goat anti-mouse IgG for 1 h at 37 °C. Horseradish peroxidase-conjugated streptavidin was added, and the sections were incubated for 30 min. Finally, the sections were exposed to fluorescence-labeled antibody and DAPI for 10 min.

### Effect of iturin A treatment on mice health

#### Weight and food intake

During the 15-day trial, the weight and food intake of mice were recorded daily.

#### Clinical biochemistry analysis of serum

At euthanasia, blood was collected after the animals had been fasted overnight. Blood samples were collected from both control and test groups via cardiac puncture under ketamine anesthesia. All blood samples were then subjected to clinical analysis of aspartate aminotransferase (AST), alanine aminotransferase (ALT), and creatinine (SCr).

### Necropsy and histopathological analysis

Mice were examined for gross lesions. Tissues and organs of interest were retained. The liver, kidneys, and spleen of all mice were weighed soon after dissection and then preserved in 4% paraformaldehyde for histopathological examination (Turki et al. 2010). Histopathological assessments were performed to evaluate viscera retained from both control and treated animals. Viscera of interest were trimmed, processed, embedded in paraffin, sectioned, and stained with H&E before their microscopic examination.

### Statistical analysis

All experiments were validated by three independent trials. Data were analyzed with GraphPad Prism version 6.0 (San Diego, CA, USA). Differences between control and treated groups were analyzed by Student t-test. A p-value below 0.05 was considered to indicate a statistically significant difference.

## Results

### Iturin A inhibits the growth of HepG2 cells by inducing apoptosis, autophagy and paraptosis

#### Inhibition of HepG2 cell growth

**Fig. 1:**
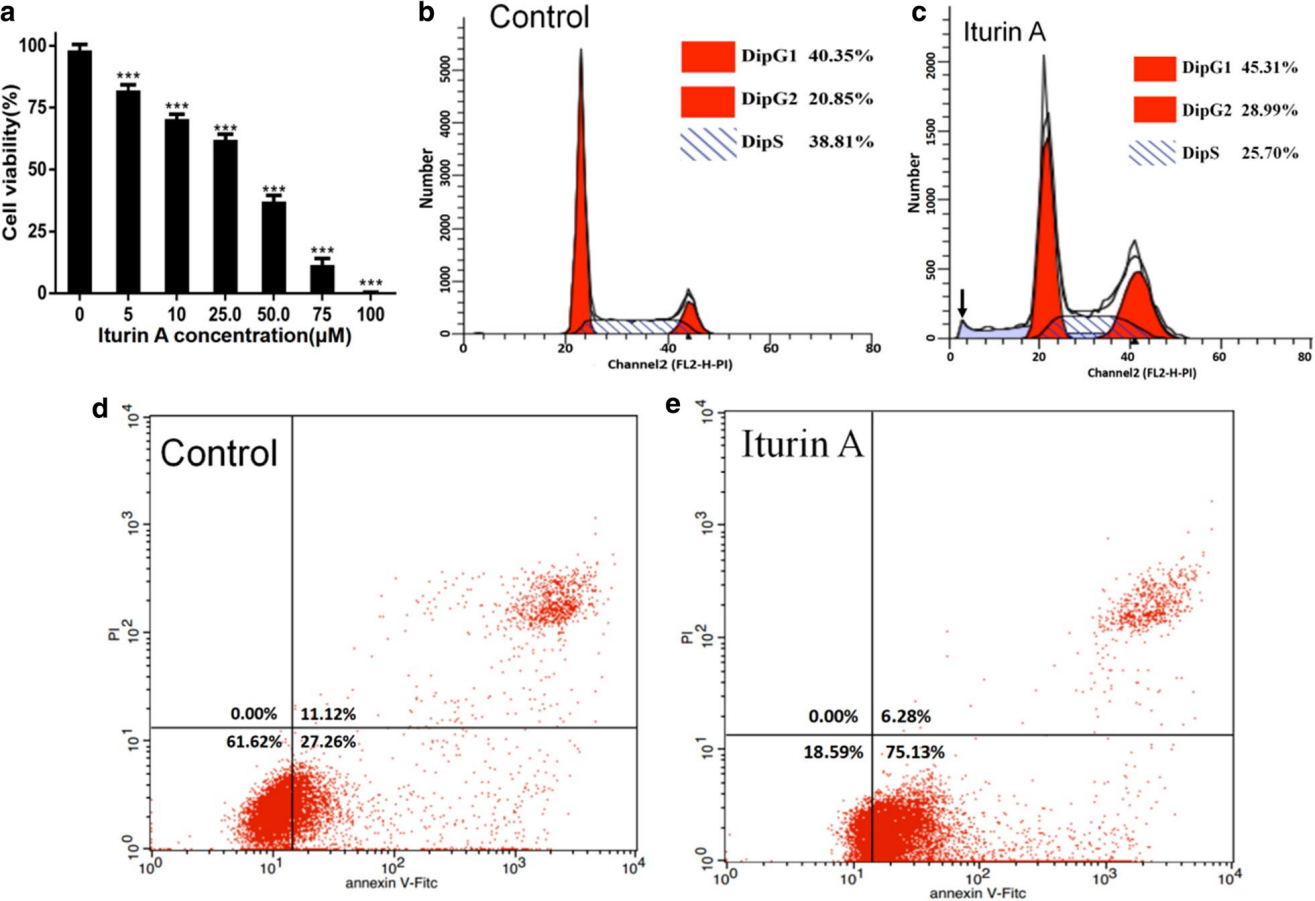
Iturin A inhibiting growth of HepG2 cell. **a** Effect of iturin A treatment on HepG2 cell viability. The inhibitory effect of iturin A on HepG2 cells was stronger than control. The higher the concentration of iturin A, the weaker the cell viability. Data are represented as the mean of each group + SD. * indicates that the iturin A effect on HepG2 was significantly different compared with the control (n = 3, p < 0.05). (**b**, **c**) Chart of cell cycle detection by flow cytometry. Iturin A treatment caused a significant increase in the portion of cells in G2/M phase and decreased the portion of cells in the S phase. The peak of apoptotic cells is indicated by the black arrow in (**c**). **d, e** Analysis of apoptotic and necrotic cell populations using flow cytometry. Iturin A treatment significantly promoted the occurrence of apoptosis, especially at the early stage in HepG2 cells

The results clearly showed iturin A treatment inhibited the growth of HepG2 cells in a concentration dependent manner. Compared with the control group, the inhibitory effect was very significant **(**Fig. 1a).

### Cell cycle arrest

Flow cytometry analysis showed that iturin A treatment caused a significant increase in the portion of cells in G2/M phase and decreased the portion of cells in the S phase, while the cells of the control group displayed relatively stable growth during the entire growth period (Fig. 1b, c).

### Induction of apoptosis in cells

The use of Annexin V-FITC in conjunction with PI allows the differentiation of apoptotic cells from early or late phase, as well as dead cells. Cells that are not stained by either annexin-V or PI are normal. Cells that are stained by annexin V-FITC only are considered early apoptotic, while cells that are stained by both annexin V-FITC and PI are late apoptotic. Fig. 1d and e shows that iturin A treatment decreased the ratio of normal cells from 61.62% to 18.59%, while it increased the total ratio of apoptotic cells from 38.38% to 81.41%. This indicated that iturin A treatment significantly promoted the occurrence of apoptosis, especially at the early stage in HepG2 cells.

### Induction of paraptosis and autophagy in cells

The defining features of paraptosis are cytoplasmic vacuoles, swollen of the mitochondria and ER. All of these were found in cells treated with iturin A. A great many of vacuoles were found in iturin A group under microscope (Additional file 1: Figure S1A, B). The image captured via TEM showed that there were a large number of cytoplasmic vacuoles in iturin A treated cells, and some of them were derived from swollen mitochondria and endoplasmic reticulum(ER) (Additional file 1: Figure S1C). All of these results illustrate that paraptosis might play an important role in the inhibitory effect of iturin A on the viability of HepG2 cells. Besides, the images captured via TEM showed that iturin A treatment caused autophagosome occurrence, which was shown in Additional file 1: Figure. S1D. This suggested that iturin A could trigger autophagy.

### Induction of ROS burst in cells

The occurrence of a ROS burst within cells is an important stress for cell growth. The increasing ROS levels inside cells could be detected by the change of DCFH-DA (without fluorescence) to fluorescent DCF in the measurement. Higher ROS levels yield stronger fluorescence intensity. As shown in Additional file 1: Figure S2, the fluorescence intensity of iturin treated cells was much stronger than that of control cells. These results indicated that iturin A treatment caused an increase of ROS within cells.

### Iturin A entering cells

In the TLC analysis, CFNSE fluctuated strongly in mobile phase, while CFNSE-iturin A hardly moved, indicating that the solvent properties of both fluorescent substances in the mobile phase are quite different. There were clearly different distances between CFNSE and CFNSE-iturin A, indicating that iturin A was successfully labelled by CFNSE. When the purified CFNSE-iturin A was mixed with HepG2 and immediately observed by fluorescence microscope, almost all cells showed fluorescence signal in the occurrence of CFNSE-iturin A, but not in the presence of CFNSE (Additional file 1: Figure S3B, C). This illustrated that iturin A could enter HepG2 cells within very short time and caused changes inside cells.

### No fatty acid chain, no activity

**Fig. 2:**
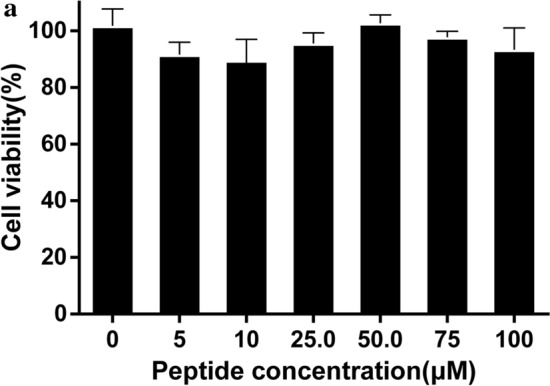
Effect of peptide in iturin A treatment on HepG2 cell viability. **a** Effect of peptide in iturin A treatment on HepG2 cell viability. The peptide could not inhibit HepG2 cell proliferation. There was no significant difference between the peptide groups and the control group (n = 3, p > 0.05)

The hydrophilic peptide and hydrophobic fatty acid chain make iturin A amphiphilic. It’s an important characteristic of iturin A. The peptide in iturin A showed no effect on HepG2 cells proliferation (Fig. 2). This indicates that fatty acid chain plays an important role in the activity of iturin A. It is speculated that the activity of iturin A is due to its amphiphilic.

### *Anticancer effect of iturin A against HCC *in vivo

#### Inhibition of tumor growth

**Fig. 3:**
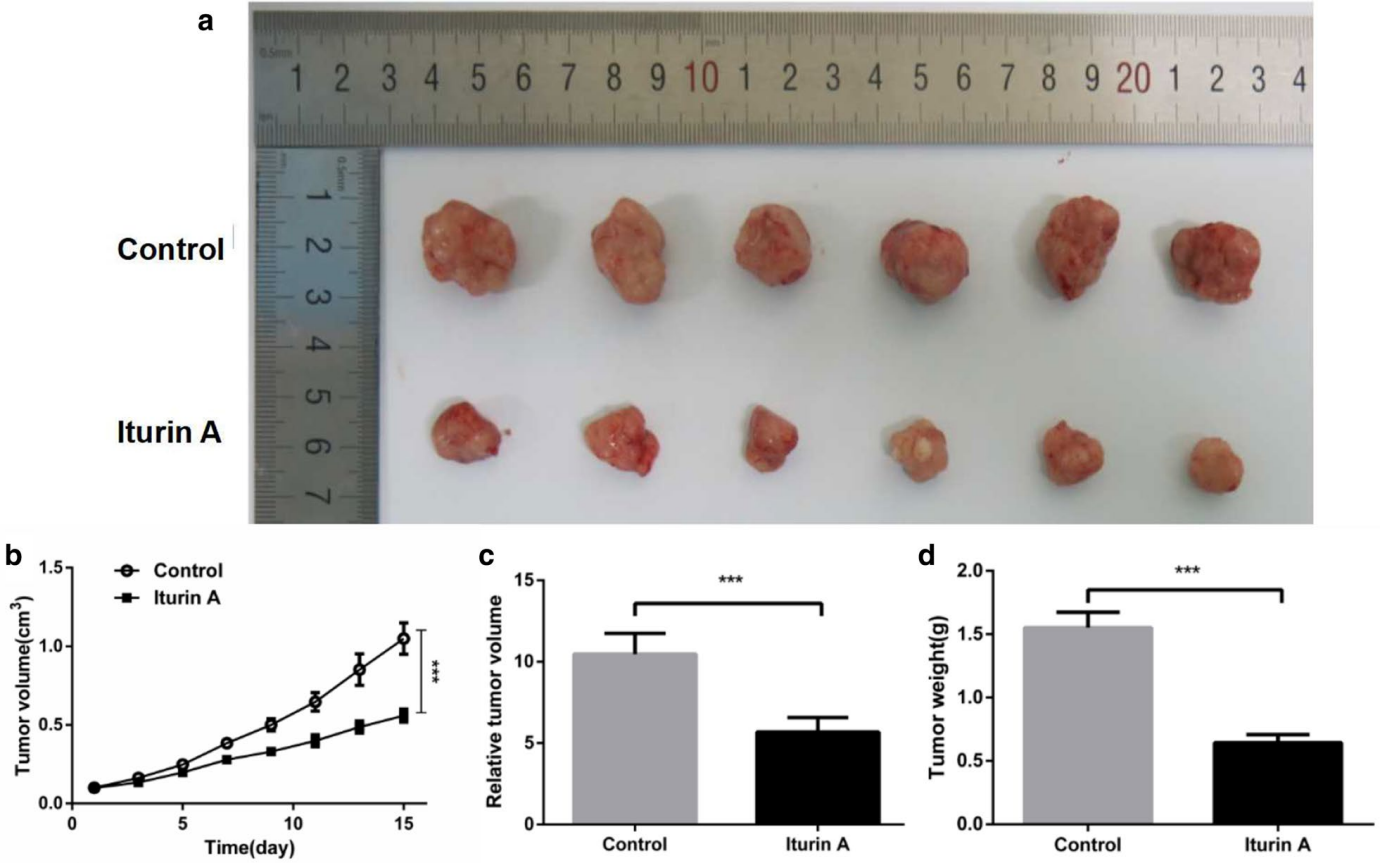
Inhibitory effect of iturin A on tumors. **a** Tumor size. The tumor size of the control group was significantly larger than that of the iturin A group. **b** Growth curve of tumor. The tumor growth rate of the control group was faster than that of the iturin A group. **c** Relative tumor volume. **d** Tumor weight. Data are represented as the mean of each group + SD. * indicates that the iturin A group was significantly different compared with the control (n = 6, p < 0.05)

As shown in Fig. 3a and b, iturin A could significantly inhibit tumor growth. Compared with the control group, the tumor volume decreased by almost 54.30% **(**Fig. 3c) and the relative tumor weight decreased by 58.55% (Fig. 3d). These results suggest that iturin A treatment could significantly inhibit the growth of HepG2 cells.

### Induction of apoptosis and autophagy in tumor cells

For apoptosis pathway analysis, the upregulated expression of bax expression was detected, while the expression of bcl-2 was decreased in the iturin A-treated tumor tissue. Furthermore, the cytochrome C amount in the cytoplasm also increased significantly (Additional file 1: Figure S4A). Therefore, it could be concluded that mitochondrial apoptosis pathway was mainly involved in the inhibitory effect of iturin A on HepG2 cell xenografts. Similar to the results of cell cultures, the activities of caspase-9 and caspase-3 were significantly enhanced in iturin A-treated tumor tissues, while activities in control groups were relatively lower (Additional file 1: Figure S4B, C).

In addition, induction of autophagy was also observed in iturin A-treated tumor tissues, which was indicated by significant increases of LC3 II expression, indicating the start of autophagy and the decrease of p62 expression related to the progression of autophagy (Additional file 1: Figure S4A). After ROS staining of frozen sections of tumor tissues, the amount of ROS was assessed. The ROS amount in iturin A-treated tumor tissue was much higher than in the control group (Additional file 1: Figure S4D).

### Improving immune microenvironment in tumor

**Fig. 4:**
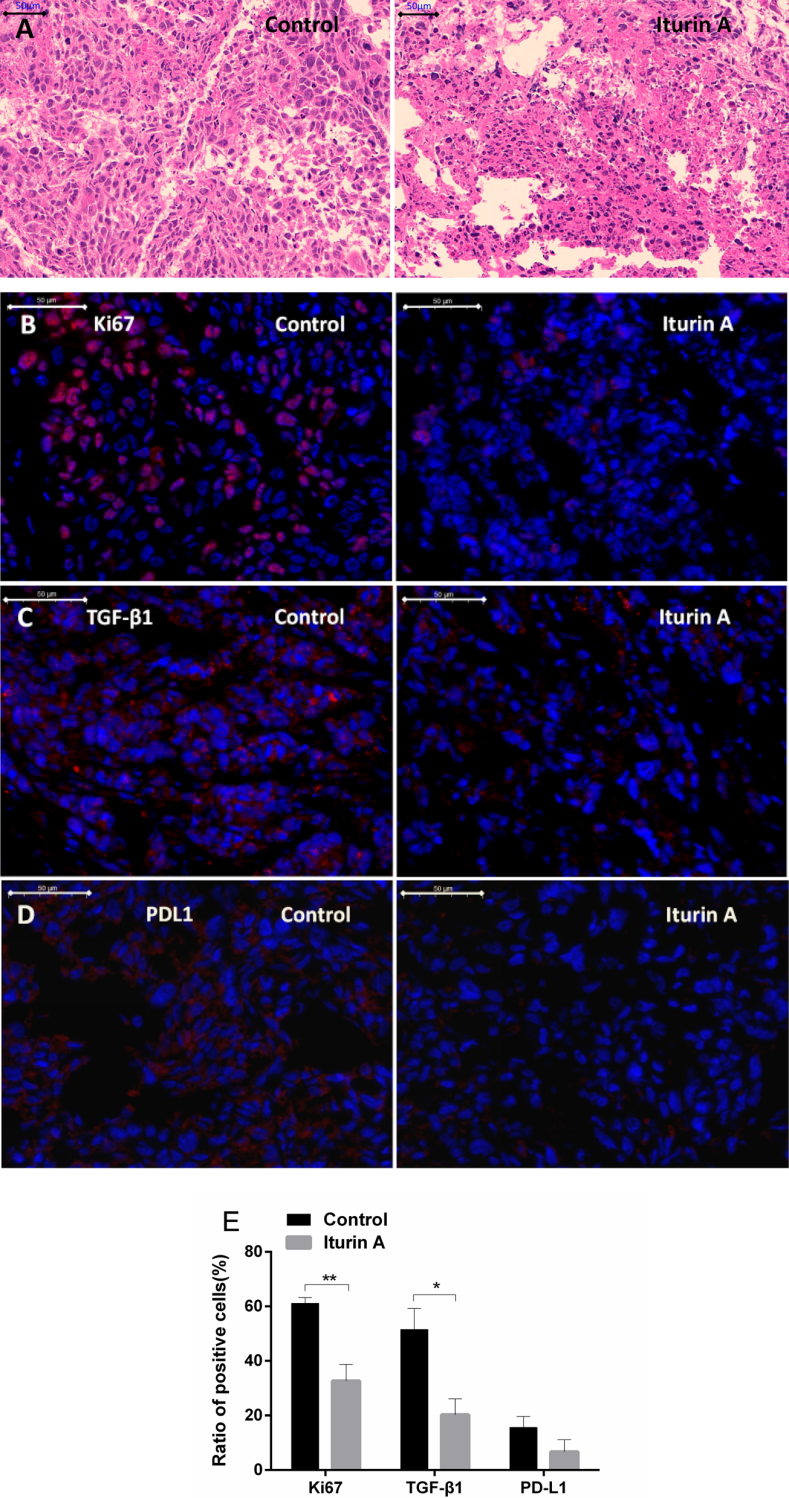
Immunohistochemistry of tumor tissue. **a** HE staining of tumor tissue. There were many tumor infiltrating lymphocytes in iturin A group. Expressions of **b** Ki67, **c** TGF-β1, and **d** PDL1 in tumor tissue. Blue indicates the nucleus, and red indicates the target proteins. The scale bar is 50 μm. **e** Ratio of positive cells. There were significant differences in the expression of Ki67 and TGF-β1. Data are represented as the mean of each group + SD. * indicates that the iturin A group was significantly different compared with the control (n = 3, p < 0.05)

Figure 4a shows many lymphocytes appeared in tumors of iturin A group. This suggests that iturin A treatment could promote lymphocyte infiltration into tumor. The tumor immune microenvironment was improved by iturin A. The results of fluorescence immunohistochemistry showed that the expression of Ki67 in tumor tissue was significantly lowered by iturin A treatment (Fig. 4b), indicating that iturin A could remarkably inhibit the growth proliferation of tumor tissues, which is consistent with the observed decrease of tumor volume and weight. TGF-β1 was significantly down-regulated by iturin A (p < 0.05). Moreover, iturin A down-regulated the expressions of PD-L1 in tumor tissues, although the effect was not significant (p > 0.05) (Fig. 4c, d). This suggested that iturin A can inhibit the immune escape of tumors.

### Effect of iturin A treatment on mice health

#### No significant influence on body weight, food intake, and viscera index in mice

**Fig. 5:**
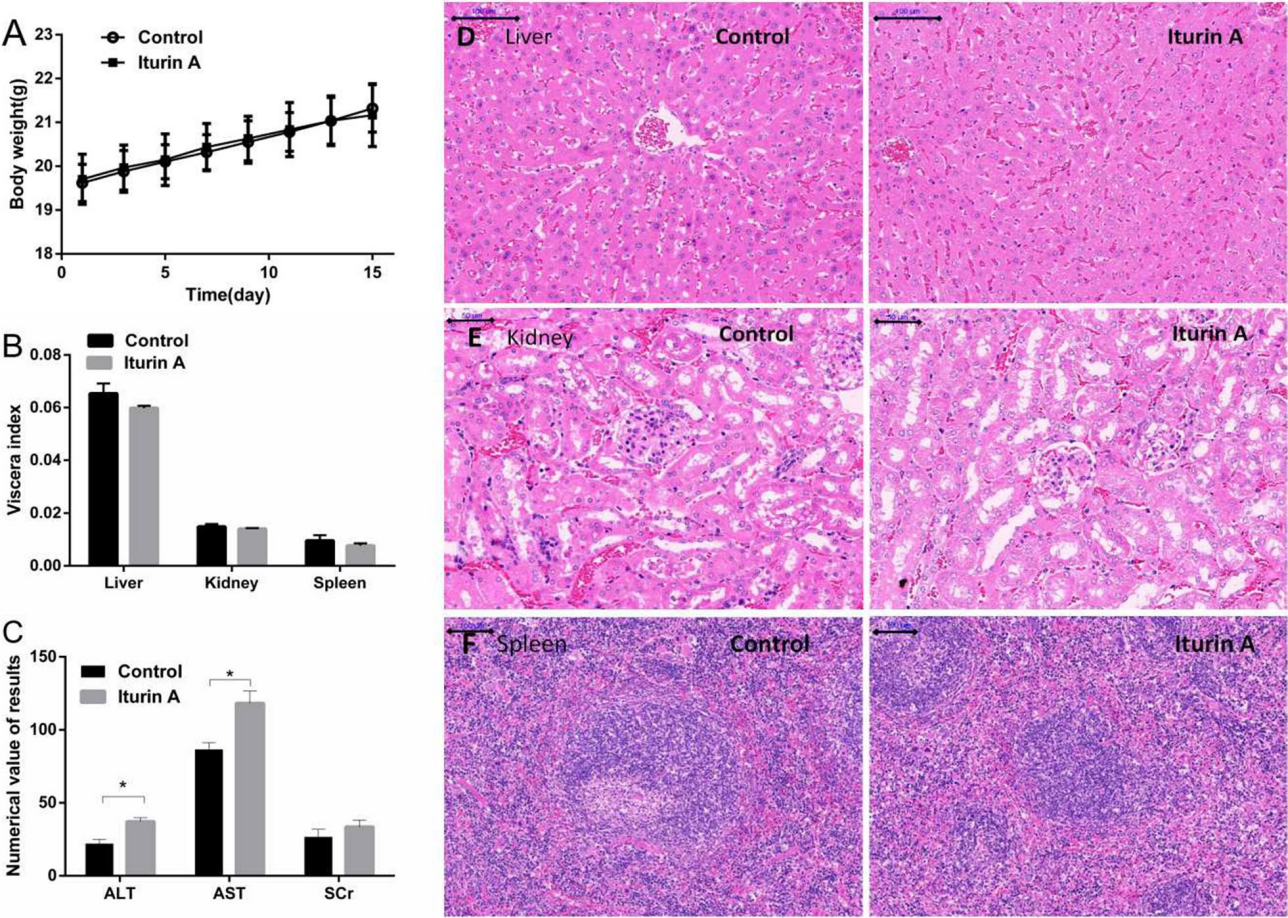
Body weight and organ examination of mice. **a** Body weight. **b** Viscera indexes. There was no significant difference in body weight and viscera indexes. **c** Activities of ALT and AST and SCr in serum. ALT and AST increased to a certain extent. **d**, **e and f** Viscera tissue sections of liver, kidney and spleen. There was congestion in hepatic sinuses in both control and treatment groups. And there was no significant difference in kidney and spleen. The scale bar is 100 μm in (**d**) and (**f**). In **e** it’s 50 μm. Data are represented as the mean of each group + SD. * indicates that the iturin A group was significantly different compared with the control (n = 6, p < 0.05)

During the experiment, no significant difference was found in the body weight between the iturin A-treated group and control group (Fig. 5a). No significant differences were found in the weight and viscera indexes of liver, spleen, and kidney between iturin A-treated and control groups (Fig. 5b). The appearance of visceras was also normal. No hepatomegaly was observed.

### Change of liver and kidney functions

At the end of the administration cycle, the serum of mice was extracted, and the ALT, AST activity, and SCr were measured. In this research, compared with the control group, serum ALT and AST in iturin A-treated mice increased significantly at the end of administration. And there was no significant difference in SCr amount (Fig. 5c). These results indicated that iturin A could interfere with the liver function of mice, but has no significant effect on the renal function.

### Chang of the structure of visceral tissue sections

Figure 5d shows a large number of red blood cells in the sinusoids of liver tissues of mice with xenograft tumors both in control and iturin A groups. Compared with the control group, the nuclei in iturin A-treated group were clearly visible, and no hepatocyte swelling, necrosis, inflammatory cell infiltration, or other phenomena were observed. In kidney tissue slices, the renal tubules and glomeruli were clearly visible and no necrotic cells or cell swelling was found in both iturin A-treated and control groups (Fig. 5e). Spleen tissue slices indicated that in both iturin A-treated and control groups, the red pulp and white pulp had clear boundaries, clear cytoplasmic nuclei, and orderly arrangement of cells without necrosis (Fig. 5f). All these results suggested that iturin A treatment was safe to kidney and spleen. Although iturin could interfere with the liver function of mice, it did not cause organic lesions.

## Discussion

This research demonstrated that iturin A from *Bacillus subtilis* had promising potential to inhibit HCC cells both in vitro and in vivo. The results of *vitro* experiments showed that iturin A could enter HCC cells and inhibit cell growth. In vivo, iturin A could retard the growth of tumor without serious side effects and improve the tumor immune microenvironment of HCC.

It has been reported that iturin A could trigger apoptosis, autophagy, and paraptosis of tumor cells (Zhao et al. 2019a). This research was consistent with previous findings. The results of *vitro* experiments showed that iturin A inhibited cell growth by disturbing the cell growth cycle, inducing apoptosis, autophagy and paraptosis. Paraptosis is one of the types of programmed cell death and is morphologically distinct from apoptosis and necrosis. The defining features of paraptosis are cytoplasmic vacuoles, swollen of the mitochondria and ER (Lee et al. 2016; Wang et al. 2012). In the simulated tumor growth environment, iturin A can only trigger apoptosis and autophagy (Zhao et al. 2019b). Induction of apoptosis and autophagy was also found in HCC xenografts, but not paraptosis. The difference of mechanisms indicated that tumor microenvironment has a great influence on the effect of iturin A. Due to factors such as vascular delivery and extracellular matrix in vivo, iturin A does not directly contact with tumor cells, which leads to changes in the mechanism.

It has been reported that lipopeptide could interact with cell membrane by hydrophobic groups, and then attack intracellular targets (Nasir and Besson 2012; Zhang et al. 2013). This research confirmed iturin A was the same as them. Amphiphilic played an important role and helped iturin A enter cells rapidly. Without fatty acid chain, iturin A lost its power. The amphiphilic maybe is the primary mechanism allowing the anticancer activity of iturin A.

In vivo experiments showed that iturin A significantly inhibited the growth of xenograft tumors in model mice. And the tumor growth inhibitory rate reached 58.55%. Compared with previous reports on breast cancer (Dey et al. 2015a), iturin A offers advantages of lower concentration and a more significant inhibitory effect on HCC xenografts.

The expression of Ki67 in cancer are positively correlated with the cancer growth rate: the higher the expression of Ki67, the stronger the proliferation activity of cancer cells (Sobecki et al. 2017). The decreased expression of Ki67 showed that iturin A could inhibit the growth of HCC cells remarkably. TGF-β1 can promote the epithelial-mesenchymal transition and activate epithelial cells to obtain the ability of invasion and migration, which plays an important role in the invasion and deterioration of tumors (Miyazono 2011). Iturin A treatment decreased the expressions of TGF-β1, in tumor tissues. These results suggested that iturin A may inhibit tumor angiogenesis and invasion. This is consistent with previous reports on other lipopeptides (Dey et al. 2017; Hajare et al. 2013; Park et al. 2013).

More importantly, iturin A treatment may reduce the immune escape ability of HCC cells. TGF-β1 plays an important role in the tumor immune microenvironment. Increased levels of TGF-β1 can block the differentiation of immature T cells into Th1 cells (Edoardo et al. 2018), and affect the antigen presenting function of dendritic cells, thus leading to the immune escape of tumor cells (Tauriello et al. 2018). PD-L1 is the ligand of PD1. PD-L1 on tumor cells binding to PD-1 on T cells can inhibit the immune activity of T cells and promote tumor escape (Oyer et al. 2018; Rensburg et al. 2018). The expression of TGF-β1 and PD-L1 in the iturin A group was decreased, which indicated that iturin A may improve the tumor immune microenvironment and inhibit the immune escape ability of HCC. Many tumor infiltrating lymphocytes in iturin A group confirmed this point.

At the same time, according to the mental state of the mice during the experiment, it can be inferred that the mice were not subjected to severe toxic or side effects of the drug. This was indicated by the bodyweight of mice. When hepatocyte necrosis occurs in animals, ALT and AST are released into the serum, resulting in a marked increase of ALT and AST activity. Therefore, the activity of serum ALT and AST is often used as a clinical auxiliary index for liver function. When the kidney is damaged, the glomerular filtration capacity decreases and the creatinine concentration in serum increases. Therefore, a high SCr can accurately reflect the renal function (Karamat et al. 2017). Iturin A treatment had no adverse effects on the kidney and spleen. Slight liver dysfunction was caused by iturin A, indicating that iturin A could cause a burden on liver metabolism. However, the appearance of liver tissue is normal without hepatomegaly, indicating that the injury by iturin A is not serious. The structure of the hepatic cord in the control group was also disordered. This may be due to the effect of xenografts.

In conclusion, *Bacillus subtilis* iturin A showed excellent anticancer activity to HCC cells in vitro and in vivo. In vitro, iturin A significantly inhibited the growth of tumor cells, induce apoptosis, autophagy and paraptosis. It could rapidly enter the cancer cells to make effects, and the inhibitory effect was dose-dependent. Amphiphilic is critical to the activity of iturin A. In vivo, iturin A significantly inhibited the growth of xenografts, and the tumor growth inhibition rate reached 58.85%. The mechanism was verified as to induce intracellular ROS burst, disrupt cell cycle, and induce apoptosis. Moreover, iturin A may promote tumor lymphocyte infiltration and improve the tumor immune microenvironment of HCC. Iturin A has no significant harm on the health of mice except slight effect on liver function. Overall, as an anticancer agent, iturin A has great application prospects, but further research is still needed.

## Supplementary Information

**Additional file1**: Figure S1 Images of optical and transmission electron microscopy (A) control HepG2 cells, (B) cells treated with iturin A, observed in optical microscope. Many cytoplasmic vacuoles were observed in iturin A treated cells. The scale bar is 100 μm. (C) and (D), ultrastructure of iturin A treated cells observed by TEM. The scale bar is 2 μm. The black arrows in (C) shows swollen mitochondria and the white arrow indicates swollen ER. Autophagosome were indicated by white arrows in (D), and the black arrows indicates lysosomes. Figure S2 ROS burst in iturin A treated cells. (A, B) Detection of ROS in cells. The fluorescence intensity of iturin treated cells was much stronger than that of control cells. The scale bar is 100 μm. (C) Fluorescence intensity of cells. There is significant difference between iturin A group and control group. Data are represented as the mean of each group + SD. * indicates that the iturin A group was significantly different compared with the control (n = 3, p < 0.05). Figure S3 Iturin A entering HepG2 cells. (A) TLC of fluorescence labelled iturin A; CFNSE has a longer migration distance. (B) CFNSE incubated with HepG2 cells; The black shadows are cells. CFNSE could not enter the cells. (C) CFNSE-Iturin A incubated with HepG2 cells. After incubation with CFNSE-iturin A, the cells showed green fluorescence. The scale bar is 100 μm. Figure S4 Apoptosis detection of tumor cells. (A) Analysis of apoptosis and autophagy. Iturin A inhibited the expression of bcl-2, up-regulated the expression of bax and promoted the release of cytochrome c. (B) Caspase-9 activity of tumor cells. (C) Caspase-3 activity of tumor cells. Iturin A treatment induced the activation of caspase 9/3. (D) Levels of ROS in tumor cells. Iturin A treatment caused ROS burst. The scale bar is 100 μm. * indicates that the iturin A group was significantly different compared with the control (n = 3, p < 0.05).

## Data Availability

The datasets generated during and/or analysed during the current study are available from the corresponding author on reasonable request.
