# Supplementary material for: Effects of Bacillus subtilis iturin A on HepG2 cells in vitro and vivo
Source: AMB Express. 2021 May 10;11:67. doi: 10.1186/s13568-021-01226-4 (PMC8110684; doi:10.1186/s13568-021-01226-4)

**Fig. S1 Images of optical and transmission electron microscopy**

**(A)** control HepG2 cells, **(B)** cells treated with iturin A, observed in optical microscope. Many cytoplasmic vacuoles were observed in iturin A treated cells. The scale bar is 100 μm. **(C)** and **(D)**, ultrastructure of iturin A treated cells observed by TEM. The scale bar is 2 μm. The black arrows in **(C)** shows swollen mitochondria and the white arrow indicates swollen ER. Autophagosome were indicated by white arrows in (D), and the black arrows indicates lysosomes.

**Fig. S2 ROS burst in iturin A treated cells**

**(A, B)** Detection of ROS in cells. The fluorescence intensity of iturin treated cells was much stronger than that of control cells. The scale bar is 100 μm. **(C)** Fluorescence intensity of cells. There is significant difference between iturin A group and control group. Data are represented as the mean of each group + SD. * indicates that the iturin A group was significantly different compared with the control (n = 3, p < 0.05).

**Fig. S3** **Iturin A entering HepG2 cells**

**(A)** TLC of fluorescence labelled iturin A; CFNSE has a longer migration distance. **(B)** CFNSE incubated with HepG2 cells; The black shadows are cells. CFNSE could not enter the cells. **(C)** CFNSE-Iturin A incubated with HepG2 cells. After incubation with CFNSE-iturin A, the cells showed green fluorescence. The scale bar is 100 μm.

**Fig. S4 Apoptosis detection of tumor cells**

**(A)** Analysis of apoptosis and autophagy. Iturin A inhibited the expression of bcl-2, up-regulated the expression of bax and promoted the release of cytochrome c. **(B)** Caspase-9 activity of tumor cells. **(C)** Caspase-3 activity of tumor cells. Iturin A treatment induced the activation of caspase 9/3. **(D)** Levels of ROS in tumor cells. Iturin A treatment caused ROS burst. The scale bar is 100 μm. * indicates that the iturin A group was significantly different compared with the control (n = 3, p < 0.05).

Fig.S1


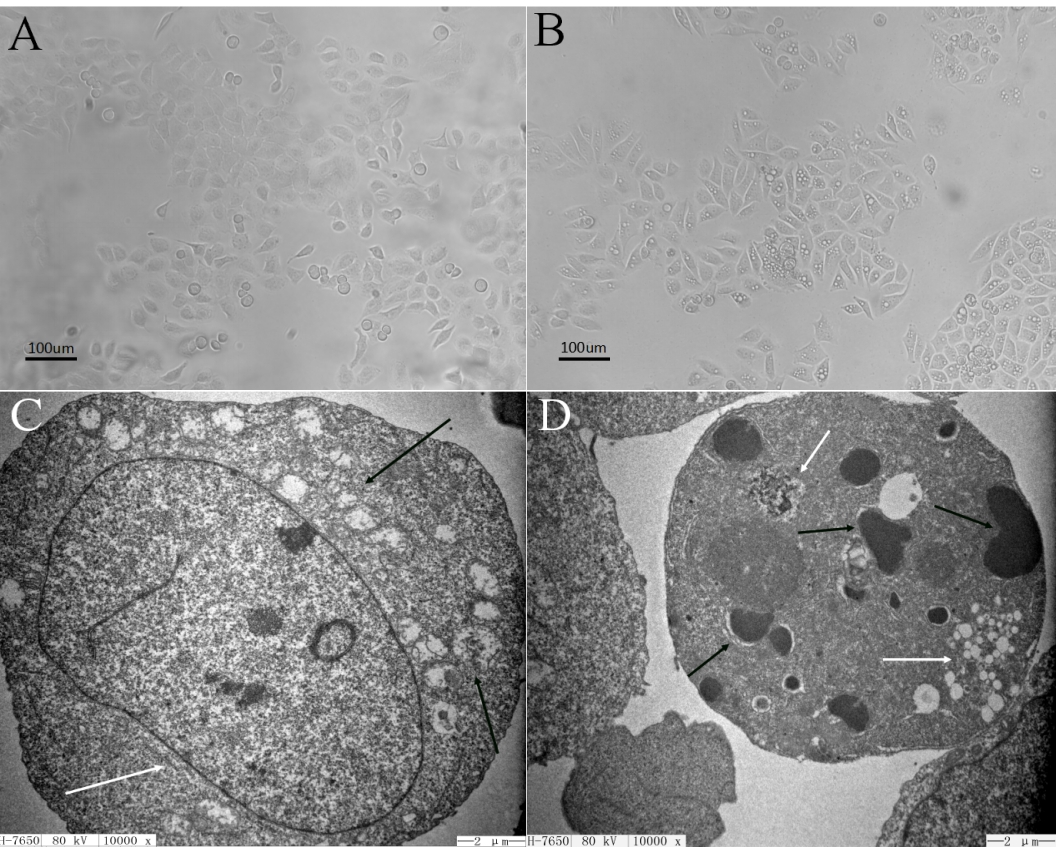


Fig.S2


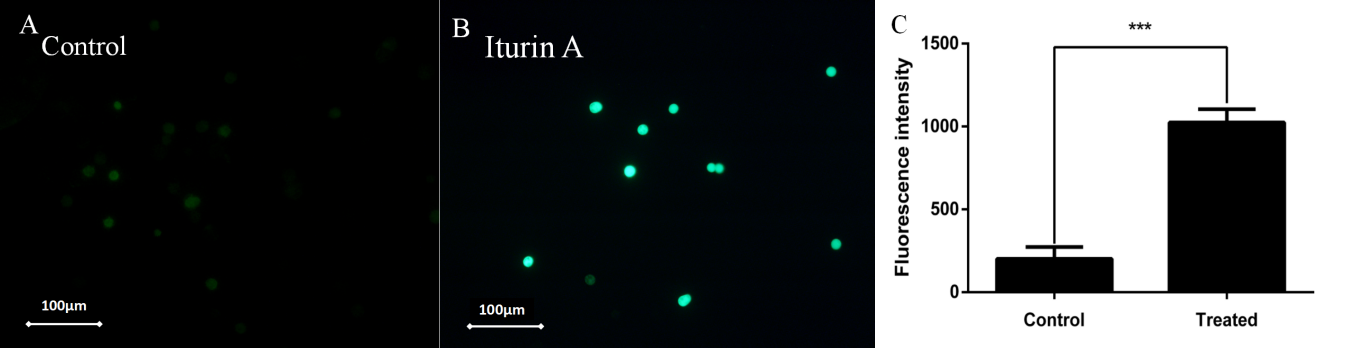


Fig.S3


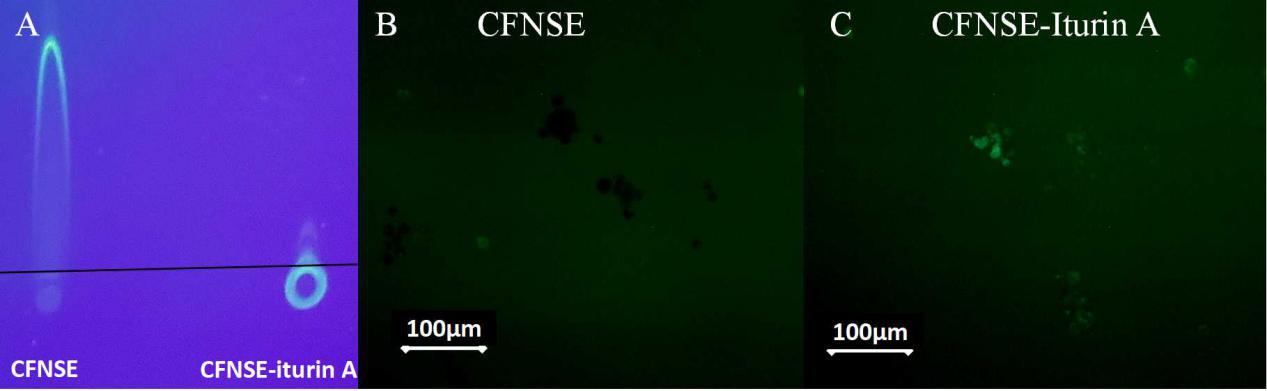


Fig.S4

INCLUDEPICTURE "C:\\Users\\Zhao\\AppData\\Local\\Temp\\ksohtml5300\\wps1.png" \* MERGEFORMATINET INCLUDEPICTURE "C:\\Users\\Zhao\\AppData\\Local\\Temp\\ksohtml5300\\wps1.png" \* MERGEFORMATINET INCLUDEPICTURE "C:\\Users\\Zhao\\AppData\\Local\\Temp\\ksohtml5300\\wps1.png" \* MERGEFORMATINET INCLUDEPICTURE "C:\\Users\\Zhao\\AppData\\Local\\Temp\\ksohtml5300\\wps1.png" \* MERGEFORMATINET
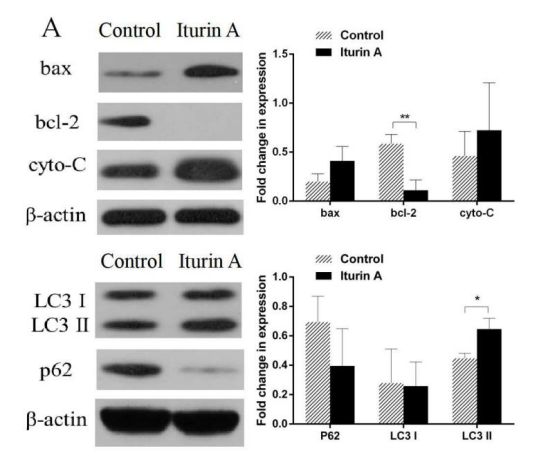
 INCLUDEPICTURE "C:\\Users\\Zhao\\AppData\\Local\\Temp\\ksohtml5300\\wps2.png" \* MERGEFORMATINET INCLUDEPICTURE "C:\\Users\\Zhao\\AppData\\Local\\Temp\\ksohtml5300\\wps2.png" \* MERGEFORMATINET INCLUDEPICTURE "C:\\Users\\Zhao\\AppData\\Local\\Temp\\ksohtml5300\\wps2.png" \* MERGEFORMATINET INCLUDEPICTURE "C:\\Users\\Zhao\\AppData\\Local\\Temp\\ksohtml5300\\wps2.png" \* MERGEFORMATINET
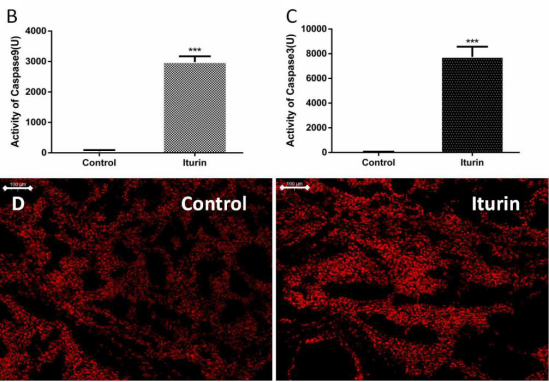

Supplement: Supplementary file 1 — Additional file1: Figure S1 Images of optical and transmission electron microscopy (A) control HepG2 cells, (B) cells treated with iturin A, observed in optical microscope. Many cytoplasmic vacuoles were observed in iturin A treated cells. The scale bar is 100 μm. (C) and (D), ultrastructure of iturin A treated cells observed by TEM. The scale bar is 2 μm. The black arrows in (C) shows swollen mitochondria and the white arrow indicates swollen ER. Autophagosome were indicated by white arrows in (D), and the black arrows indicates lysosomes. Figure S2 ROS burst in iturin A treated cells. (A, B) Detection of ROS in cells. The fluorescence intensity of iturin treated cells was much stronger than that of control cells. The scale bar is 100 μm. (C) Fluorescence intensity of cells. There is significant difference between iturin A group and control group. Data are represented as the mean of each group + SD. * indicates that the iturin A group was significantly different compared with the control (n = 3, p < 0.05). Figure S3 Iturin A entering HepG2 cells. (A) TLC of fluorescence labelled iturin A; CFNSE has a longer migration distance. (B) CFNSE incubated with HepG2 cells; The black shadows are cells. CFNSE could not enter the cells. (C) CFNSE-Iturin A incubated with HepG2 cells. After incubation with CFNSE-iturin A, the cells showed green fluorescence. The scale bar is 100 μm. Figure S4 Apoptosis detection of tumor cells. (A) Analysis of apoptosis and autophagy. Iturin A inhibited the expression of bcl-2, up-regulated the expression of bax and promoted the release of cytochrome c. (B) Caspase-9 activity of tumor cells. (C) Caspase-3 activity of tumor cells. Iturin A treatment induced the activation of caspase 9/3. (D) Levels of ROS in tumor cells. Iturin A treatment caused ROS burst. The scale bar is 100 μm. * indicates that the iturin A group was significantly different compared with the control (n = 3, p < 0.05). [file 13568_2021_1226_MOESM1_ESM.doc]
